# (Butane-1,3-diyne-1,4-diyl)bis­(tri­isopropyl­silane)

**DOI:** 10.1107/S160053681002725X

**Published:** 2010-07-17

**Authors:** Muhammad Raza Shah, Seik Weng Ng

**Affiliations:** a H.E.J. Research Institute of Chemistry, International Center for Chemical and Biological Sciences, University of Karachi, Karachi 75270, Pakistan; b Department of Chemistry, University of Malaya, 50603 Kuala Lumpur, Malaysia

## Abstract

The mol­ecule of the title compound, C_22_H_42_Si_2_, lies on a center of inversion, and the triisopropyl­silyl groups are staggered.

## Related literature

For the crystal structures of the trimethyl and tris-*tert*-butyl analogs, see: Bruckmann & Krüger (1997 ▶); Vitze *et al.* (2009 ▶).
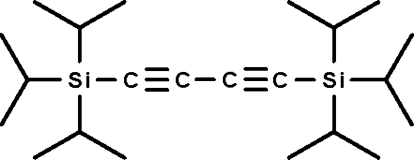


## Experimental

### 

#### Crystal data

C_22_H_42_Si_2_
*M* *_r_* = 362.74
Triclinic, 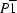

*a* = 7.1213 (10) Å
*b* = 7.9057 (11) Å
*c* = 10.6937 (14) Å
α = 89.139 (2)°
β = 81.823 (2)°
γ = 79.449 (2)°
*V* = 585.81 (14) Å^3^
*Z* = 1
Mo *K*α radiation
μ = 0.15 mm^−1^
*T* = 100 K
0.40 × 0.10 × 0.10 mm

#### Data collection

Bruker SMART APEX diffractometer
Absorption correction: multi-scan (*SADABS*; Sheldrick, 1996 ▶) *T*_min_ = 0.941, *T*_max_ = 0.985
5560 measured reflections
2674 independent reflections
2190 reflections with *I* > 2σ(*I*)
*R*_int_ = 0.036

#### Refinement

*R*[*F* ^2^ > 2σ(*F* ^2^)] = 0.042
*wR*(*F* ^2^) = 0.116
*S* = 1.06
2674 reflections
115 parameters
H-atom parameters constrained
Δρ_max_ = 0.38 e Å^−3^
Δρ_min_ = −0.34 e Å^−3^

### 

Data collection: *APEX2* (Bruker, 2009 ▶); cell refinement: *SAINT* (Bruker, 2009 ▶); data reduction: *SAINT*; program(s) used to solve structure: *SHELXS97* (Sheldrick, 2008 ▶); program(s) used to refine structure: *SHELXL97* (Sheldrick, 2008 ▶); molecular graphics: *X-SEED* (Barbour, 2001 ▶); software used to prepare material for publication: *publCIF* (Westrip, 2010 ▶).

## Supplementary Material

Crystal structure: contains datablocks global, I. DOI: 10.1107/S160053681002725X/jh2179sup1.cif

Structure factors: contains datablocks I. DOI: 10.1107/S160053681002725X/jh2179Isup2.hkl

Additional supplementary materials:  crystallographic information; 3D view; checkCIF report

## Figures and Tables

**Fig. 1.**
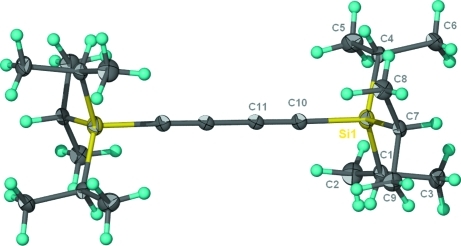
Thermal ellipsoid plot (Barbour, 2001) of C24H42Si2 at the 70% probability level; hydrogen atoms are drawn as spheres of arbitrary radius.

## supplementary crystallographic information

### Comment

The compound (Scheme I) was obtained in an unsuccessful attempt at the Sonogoshira coupling of 2,9-dichloro-1,10-phenanthroline with trisisopropylsilylacetylene. The carbon–carbon triple-bond is 1.210 (2) Å long; the distance is indistinguisahble from that [1.208 (3) Å] for bis(trimethylsilyl)acetylene (Bruckmann & Krüger, 1997) as well as that [1.22 (2) Å] found in the *t*-butyl analog (Vitze *et al.* (2009). The molecule lies on a center of inversion, and the trisisopropylsilyl groups are staggered (Fig. 1).

### Experimental

Copper(I) iodide (70 mg, 0.36 mmol) and dichlorobis(triphenylphosphine)palladium (10 mg, 0.014 mmol) were added to a pyridine solution (10 ml) of triisopropylsilylacetylene (440 mg, 2.4 mmol) and 2,9-dichloro-1,10-phenanthroline (200 mg, 0.8 mmol). The solution was stirred for 4 h. The pyridine was removed under vacuum and the residue dissolved in dichloromethane (10 ml). The solution was washed with 2 N hydrochloric acid (10 ml). The solvent was evaporated and the solid recrystallized from dichloromethane to afford colorless crystals.

### Refinement

Carbon-bound H-atoms were placed in calculated positions [C–H 0.98–1.00 Å, *U*(H) 1.2–1.5*U*(C)] and were included in the refinement in the riding model approximation.

### Crystal data

**Table:**

C_22_H_42_Si_2_	*Z* = 1
*M**_r_* = 362.74	*F*(000) = 202
Triclinic, *P*1	*D*_x_ = 1.028 Mg m^−^^3^
Hall symbol: -P 1	Mo *K*α radiation, λ = 0.71073 Å
*a* = 7.1213 (10) Å	Cell parameters from 1787 reflections
*b* = 7.9057 (11) Å	θ = 2.6–27.7°
*c* = 10.6937 (14) Å	µ = 0.15 mm^−^^1^
α = 89.139 (2)°	*T* = 100 K
β = 81.823 (2)°	Prism, colorless
γ = 79.449 (2)°	0.40 × 0.10 × 0.10 mm
*V* = 585.81 (14) Å^3^	

### Data collection

**Table:**

Bruker SMART APEX diffractometer	2674 independent reflections
Radiation source: fine-focus sealed tube	2190 reflections with *I* > 2σ(*I*)
graphite	*R*_int_ = 0.036
ω scans	θ_max_ = 27.5°, θ_min_ = 1.9°
Absorption correction: multi-scan (*SADABS*; Sheldrick, 1996)	*h* = −9→9
*T*_min_ = 0.941, *T*_max_ = 0.985	*k* = −10→10
5560 measured reflections	*l* = −13→13

### Refinement

**Table:**

Refinement on *F*^2^	Primary atom site location: structure-invariant direct methods
Least-squares matrix: full	Secondary atom site location: difference Fourier map
*R*[*F*^2^ > 2σ(*F*^2^)] = 0.042	Hydrogen site location: inferred from neighbouring sites
*wR*(*F*^2^) = 0.116	H-atom parameters constrained
*S* = 1.06	*w* = 1/[σ^2^(*F*_o_^2^) + (0.0577*P*)^2^ + 0.057*P*] where *P* = (*F*_o_^2^ + 2*F*_c_^2^)/3
2674 reflections	(Δ/σ)_max_ = 0.001
115 parameters	Δρ_max_ = 0.38 e Å^−^^3^
0 restraints	Δρ_min_ = −0.34 e Å^−^^3^

### Fractional atomic coordinates and isotropic or equivalent isotropic displacement parameters (Å^2^)

**Table:**

	*x*	*y*	*z*	*U*_iso_*/*U*_eq_	
Si1	0.75095 (6)	0.77829 (5)	0.27093 (4)	0.01479 (15)	
C1	0.7369 (2)	0.7104 (2)	0.10482 (15)	0.0195 (4)	
H1	0.7950	0.5852	0.0980	0.023*	
C2	0.5337 (3)	0.7257 (2)	0.07088 (18)	0.0295 (4)	
H2A	0.5401	0.6716	−0.0118	0.044*	
H2B	0.4745	0.8475	0.0677	0.044*	
H2C	0.4559	0.6679	0.1349	0.044*	
C3	0.8628 (3)	0.8009 (2)	0.00649 (16)	0.0269 (4)	
H3A	0.8642	0.7528	−0.0776	0.040*	
H3B	0.9947	0.7831	0.0271	0.040*	
H3C	0.8096	0.9244	0.0074	0.040*	
C4	0.6408 (2)	1.0072 (2)	0.31850 (16)	0.0185 (4)	
H4	0.6446	1.0147	0.4113	0.022*	
C5	0.4277 (3)	1.0599 (2)	0.30187 (18)	0.0264 (4)	
H5A	0.3746	1.1712	0.3438	0.040*	
H5B	0.3558	0.9728	0.3395	0.040*	
H5C	0.4165	1.0693	0.2116	0.040*	
C6	0.7578 (3)	1.1395 (2)	0.25767 (18)	0.0282 (4)	
H6A	0.7061	1.2526	0.2972	0.042*	
H6B	0.7489	1.1459	0.1671	0.042*	
H6C	0.8932	1.1044	0.2699	0.042*	
C7	1.0093 (2)	0.7272 (2)	0.30172 (16)	0.0178 (4)	
H7	1.0807	0.8091	0.2514	0.021*	
C8	1.0237 (3)	0.7550 (2)	0.44066 (17)	0.0233 (4)	
H8A	1.1598	0.7363	0.4527	0.035*	
H8B	0.9585	0.6737	0.4923	0.035*	
H8C	0.9620	0.8730	0.4662	0.035*	
C9	1.1074 (2)	0.5444 (2)	0.25796 (17)	0.0233 (4)	
H9A	1.2368	0.5190	0.2827	0.035*	
H9B	1.1173	0.5358	0.1658	0.035*	
H9C	1.0307	0.4617	0.2973	0.035*	
C10	0.6199 (2)	0.64130 (19)	0.37986 (15)	0.0161 (3)	
C11	0.5440 (2)	0.55159 (19)	0.45656 (14)	0.0152 (3)	

### Atomic displacement parameters (Å^2^)

**Table:**

	*U*^11^	*U*^22^	*U*^33^	*U*^12^	*U*^13^	*U*^23^
Si1	0.0163 (3)	0.0141 (2)	0.0141 (3)	−0.00465 (17)	−0.00050 (17)	0.00352 (16)
C1	0.0248 (9)	0.0177 (8)	0.0167 (9)	−0.0068 (7)	−0.0015 (7)	0.0022 (6)
C2	0.0319 (11)	0.0371 (11)	0.0229 (10)	−0.0123 (9)	−0.0080 (8)	0.0002 (8)
C3	0.0355 (11)	0.0301 (10)	0.0170 (9)	−0.0146 (8)	0.0009 (8)	0.0004 (7)
C4	0.0228 (9)	0.0173 (8)	0.0146 (8)	−0.0041 (7)	0.0006 (7)	0.0028 (6)
C5	0.0269 (10)	0.0230 (9)	0.0266 (10)	0.0029 (7)	−0.0043 (8)	0.0015 (7)
C6	0.0361 (11)	0.0163 (8)	0.0302 (11)	−0.0070 (8)	0.0051 (9)	0.0005 (7)
C7	0.0171 (8)	0.0166 (8)	0.0200 (9)	−0.0055 (6)	−0.0010 (7)	0.0022 (6)
C8	0.0192 (9)	0.0254 (9)	0.0263 (10)	−0.0034 (7)	−0.0077 (7)	0.0010 (7)
C9	0.0198 (9)	0.0215 (9)	0.0273 (10)	−0.0009 (7)	−0.0029 (7)	0.0016 (7)
C10	0.0159 (8)	0.0150 (7)	0.0174 (9)	−0.0017 (6)	−0.0041 (7)	0.0021 (6)
C11	0.0148 (8)	0.0142 (7)	0.0168 (9)	−0.0010 (6)	−0.0049 (7)	0.0001 (6)

### Geometric parameters (Å, °)

**Table:**

Si1—C10	1.8504 (16)	C5—H5B	0.9800
Si1—C4	1.8822 (17)	C5—H5C	0.9800
Si1—C1	1.8848 (17)	C6—H6A	0.9800
Si1—C7	1.8849 (17)	C6—H6B	0.9800
C1—C2	1.524 (2)	C6—H6C	0.9800
C1—C3	1.538 (2)	C7—C8	1.526 (2)
C1—H1	1.0000	C7—C9	1.533 (2)
C2—H2A	0.9800	C7—H7	1.0000
C2—H2B	0.9800	C8—H8A	0.9800
C2—H2C	0.9800	C8—H8B	0.9800
C3—H3A	0.9800	C8—H8C	0.9800
C3—H3B	0.9800	C9—H9A	0.9800
C3—H3C	0.9800	C9—H9B	0.9800
C4—C5	1.533 (2)	C9—H9C	0.9800
C4—C6	1.535 (2)	C10—C11	1.210 (2)
C4—H4	1.0000	C11—C11^i^	1.385 (3)
C5—H5A	0.9800		
			
C10—Si1—C4	106.04 (7)	C4—C5—H5B	109.5
C10—Si1—C1	107.42 (7)	H5A—C5—H5B	109.5
C4—Si1—C1	117.06 (8)	C4—C5—H5C	109.5
C10—Si1—C7	105.69 (7)	H5A—C5—H5C	109.5
C4—Si1—C7	110.41 (7)	H5B—C5—H5C	109.5
C1—Si1—C7	109.52 (8)	C4—C6—H6A	109.5
C2—C1—C3	110.85 (14)	C4—C6—H6B	109.5
C2—C1—Si1	115.47 (12)	H6A—C6—H6B	109.5
C3—C1—Si1	111.67 (11)	C4—C6—H6C	109.5
C2—C1—H1	106.0	H6A—C6—H6C	109.5
C3—C1—H1	106.0	H6B—C6—H6C	109.5
Si1—C1—H1	106.0	C8—C7—C9	110.89 (14)
C1—C2—H2A	109.5	C8—C7—Si1	111.21 (11)
C1—C2—H2B	109.5	C9—C7—Si1	111.98 (11)
H2A—C2—H2B	109.5	C8—C7—H7	107.5
C1—C2—H2C	109.5	C9—C7—H7	107.5
H2A—C2—H2C	109.5	Si1—C7—H7	107.5
H2B—C2—H2C	109.5	C7—C8—H8A	109.5
C1—C3—H3A	109.5	C7—C8—H8B	109.5
C1—C3—H3B	109.5	H8A—C8—H8B	109.5
H3A—C3—H3B	109.5	C7—C8—H8C	109.5
C1—C3—H3C	109.5	H8A—C8—H8C	109.5
H3A—C3—H3C	109.5	H8B—C8—H8C	109.5
H3B—C3—H3C	109.5	C7—C9—H9A	109.5
C5—C4—C6	110.60 (14)	C7—C9—H9B	109.5
C5—C4—Si1	114.57 (11)	H9A—C9—H9B	109.5
C6—C4—Si1	113.60 (11)	C7—C9—H9C	109.5
C5—C4—H4	105.7	H9A—C9—H9C	109.5
C6—C4—H4	105.7	H9B—C9—H9C	109.5
Si1—C4—H4	105.7	C11—C10—Si1	175.41 (14)
C4—C5—H5A	109.5	C10—C11—C11^i^	179.4 (2)
			
C10—Si1—C1—C2	59.25 (14)	C1—Si1—C4—C6	−72.44 (15)
C4—Si1—C1—C2	−59.79 (14)	C7—Si1—C4—C6	53.75 (15)
C7—Si1—C1—C2	173.58 (12)	C10—Si1—C7—C8	−56.07 (12)
C10—Si1—C1—C3	−172.91 (12)	C4—Si1—C7—C8	58.19 (13)
C4—Si1—C1—C3	68.05 (14)	C1—Si1—C7—C8	−171.50 (11)
C7—Si1—C1—C3	−58.59 (14)	C10—Si1—C7—C9	68.62 (13)
C10—Si1—C4—C5	−63.77 (14)	C4—Si1—C7—C9	−177.12 (11)
C1—Si1—C4—C5	56.00 (14)	C1—Si1—C7—C9	−46.81 (13)
C7—Si1—C4—C5	−177.80 (12)	C1—Si1—C10—C11	142.3 (18)
C10—Si1—C4—C6	167.78 (12)		

Symmetry codes: (i) −*x*+1, −*y*+1, −*z*+1.
